# Western gorilla space use suggests territoriality

**DOI:** 10.1038/s41598-020-60504-6

**Published:** 2020-03-12

**Authors:** Robin E. Morrison, Jacob C. Dunn, Germán Illera, Peter D. Walsh, Magdalena Bermejo

**Affiliations:** 1 Biological Anthropology, University of Cambridge, Cambridge, UK; 2 SPAC Scientific Field Station Network, GgmbH, Bielefeld, Germany; 3 Behavioural Ecology Research Group, Anglia Ruskin University, Cambridge, UK; 4 Department of Cognitive Biology, University of Vienna, Vienna, Austria; 5 Apes Incorporated, Palo Alto, USA; 6 Ecology and Environmental Sciences, University of Barcelona, Barcelona, Spain; 7 Karisoke Research Center, The Dian Fossey Gorilla Fund, Musanze, Rwanda

**Keywords:** Behavioural ecology, Evolutionary ecology, Biological anthropology, Social evolution

## Abstract

The evolutionary origins of how modern humans share and use space are often modelled on the territorial-based violence of chimpanzees, with limited comparison to other apes. Gorillas are widely assumed to be non-territorial due to their large home ranges, extensive range overlap, and limited inter-group aggression. Using large-scale camera trapping, we monitored western gorillas in Republic of Congo across 60 km^2^. Avoidance patterns between groups were consistent with an understanding of the “ownership” of specific regions, with greater avoidance of their neighbours the closer they were to their neighbours’ home range centres. Groups also avoided larger groups’ home ranges to a greater extent, consistent with stronger defensive responses from more dominant groups. Our results suggest that groups may show territoriality, defending core regions of their home ranges against neighbours, and mirror patterns common across human evolution, with core areas of resident dominance and larger zones of mutual tolerance. This implies western gorillas may be a key system for understanding how humans have evolved the capacity for extreme territorial-based violence and warfare, whilst also engaging in the strong affiliative inter-group relationships necessary for large-scale cooperation.

## Introduction

Understanding how neighbours use and share space is fundamental to understanding a species’ large-scale social system^1^. Patterns of space use can have considerable impacts on the likelihood of neighbours encountering one another, and the location of such encounters can greatly influence the behaviour shown when they meet^2–4^. One key example of this is in species showing territoriality, where rates of aggression shown to out-group individuals can differ drastically depending on the location relative to the group’s territory^5–8^.

Territories are commonly defined as regions of a home range that are actively defended against intruders to enable exclusive use by the individual or social unit^9^. However, there is considerable variation in how the term has been used in different study systems^10^ and broader definitions of territoriality also include areas of priority use^1,11^, for example, through site-specific dominance^8,12^. Territories can be defended using physical aggression or advertised using less costly alternatives such as scent marking, calls or displays; with a broad diversity of territorial behaviors observed both within and among species^11^, including humans^13^. It has been increasingly suggested that defining territoriality as a binary trait cannot explain the full diversity of territorial behaviours observed^1,11,14–16^. Territoriality may be better described by a continuum from extreme territoriality where neighbouring conspecifics impose a hard boundary on movement, to species with more flexible territory boundaries such as the black bear where territoriality can vary, geographically and temporally^11^, through to non-territorial species in which the ranges of neighbouring conspecifics do not constrain an individual or group’s movement patterns^14^. Whilst this may stretch more traditional definitions of territoriality, what should not be overlooked is the wide variation in the extent to which the location of neighbours’ home ranges influence patterns of avoidance and aggression. Regardless of how this is defined, it is fundamental to how neighbouring conspecifics share and use space and a better understanding of this variation is key for the study of animal social systems.

Territoriality is widespread among apes, having been observed in gibbons (*Hylobatidae*)^17^, chimpanzees (*Pan troglodytes*)^18,19^ and humans (*Homo sapiens*)^20^, although is reported to be absent in orangutans (*Pongo*)^21,22^ and at least some populations of bonobos (*Pan paniscus*)^4,23^. Gorillas are widely assumed to be non-territorial due to the presence of home range overlap and observations of tolerant between-group interactions^24–26^. The presence of territoriality in primates is correlated with a group’s ability to patrol it’s home range on a daily basis^15,27^. However, the large sizes of gorilla groups’ home ranges suggest that they are not defendable in their entirety, as the home ranges observed in habituated western gorilla groups (11–18 km^2^) are far larger than the average daily path lengths of 1.7–2 km^24,28–30^. Considerable range overlap between gorilla groups^24^ indicates that territoriality under the definition of exclusive use of defended space^9^ cannot be present across the entirety of their home ranges. However, this does not rule out the presence of territoriality, even under its narrowest of definitions, as territories represent only a subsection of the total home range^9^, leaving the possibility of home range overlap and tolerant interactions outside the territory.

Mountain gorilla (*Gorilla beringei beringei*) groups have been found to use the core areas of their home ranges almost exclusively and avoid neighbouring groups^25,31^. They have also been found to reduce their home ranges in response to increased population density^14^, an avoidance behaviour typically observed in territorial species, demonstrating that the presence of neighbouring groups represent a social barrier constraining movement patterns. This led the authors to suggest that mountain gorillas may be intermediate on a continuum between non-territoriality and territoriality^14,25^.

**Figure 1:**
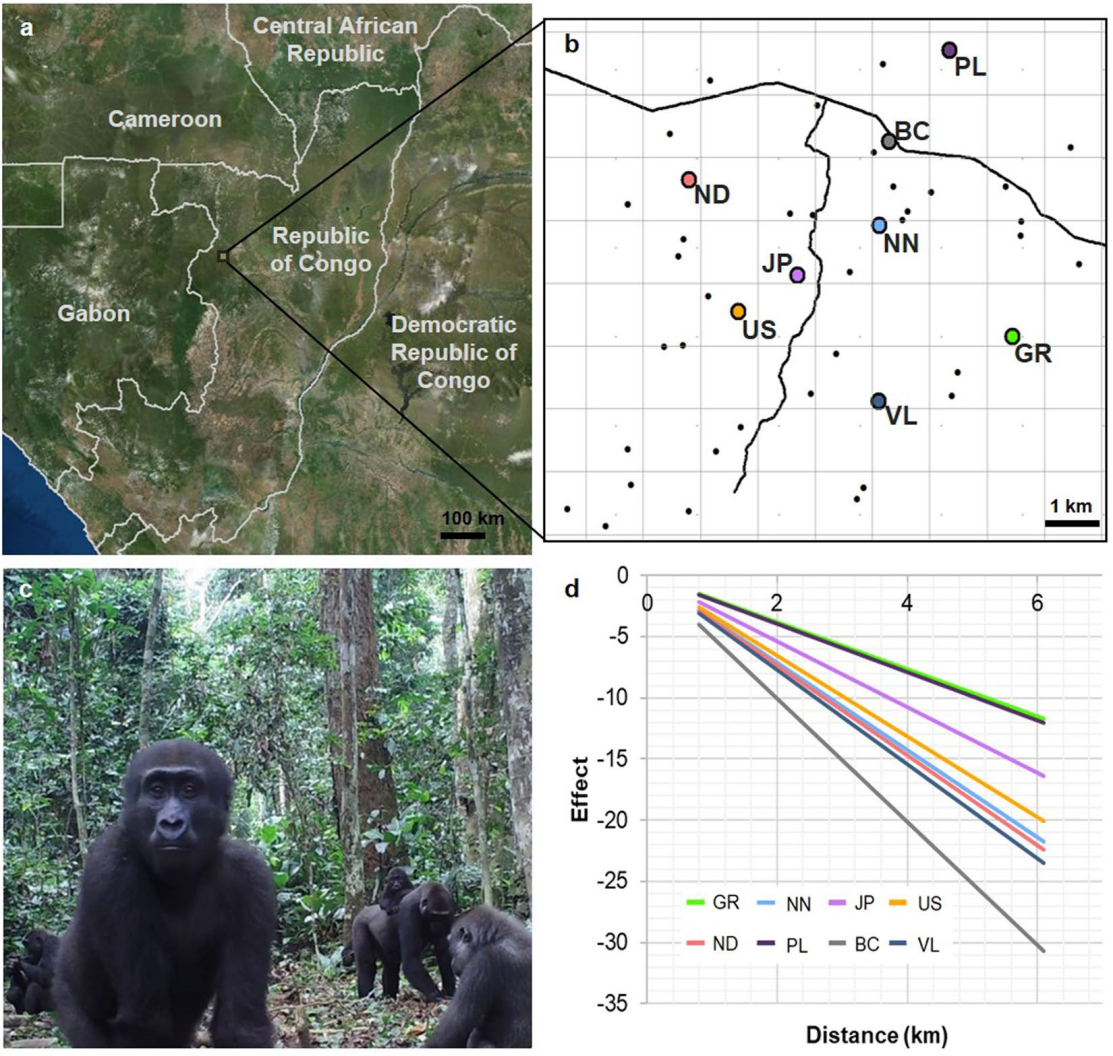
Reconstructing ranging patterns by camera trapping. (**a**) Location of Ngaga Research Site within Republic of Congo with world imagery base map, created in arcGIS version 10.4 (www.esri.com sources: Esri, DigitalGlobe, GeoEye, i-cubed, USDA FSA, USGS, AEX, Getmapping, Aerogrid, IGN, IGP, swisstopo, and the GIS User Community). (**b**) Location of camera traps deployed across the research site with estimated home range centroids for the eight focal groups (indicated by large coloured dots) from the baseline model, with 1 km^2^ grid overlaid. Camera trap locations indicated by small black dots, road indicated by black line. (**c**) Example camera trap image from which gorilla groups and individuals were identified. (**d**) The predicted presence of all eight focal gorilla groups with distance from their home range centroid.

Far less is known about the space use patterns of the western species of gorilla (*Gorilla gorilla*), in which their dense forest habitat and slow habituation process has prevented the simultaneous monitoring of more than a small number of groups through direct observation^24,28–30^. Therefore, to investigate space use in western lowland gorillas (*Gorilla gorilla gorilla*), we sampled a 60 km^2^ area in the peripheral regions of Odzala-Kokoua National Park, Republic of Congo, using automated camera traps at 36 naturally occurring feeding hotspots (Fig. 1). These represented permanent, non-seasonal resources (Supplementary Fig. S1) at which gorillas would spend adequate time feeding to enable individual identification by non-invasive camera trap monitoring. Between January 2015 and July 2016, 568 distinct gorilla visits were recorded, including those from a total of 24 identified groups. The group or individual present was identified in 76.9% of visits (Table S1). Eight groups (referred to as focal groups) were recorded on at least 10 separate occasions, in 3 or more locations, enabling their ranging patterns to be modelled (Supplementary Figs. S2 and S3).

A key component of many definitions of territoriality includes the presence of active exclusion of neighbours through the defence of space. However, the current lack of ranging and behavioural data from a large number of neighbouring, habituated western gorilla groups prevents the possibility of investigating such a behaviour directly. We therefore investigated patterns of avoidance, modelling how the ranging behavior of 8 focal groups recorded via camera trapping was influenced by both the presence of conspecifics and the location of those conspecifics’ home ranges.

A variety of reasons for the avoidance of neighbouring groups in gorillas have been suggested, including mating competition and feeding competition (both contest and scramble)^14,25^. Fundamentally, explanations for avoidance can be divided into 1) reasons to minimize interacting with other groups, and therefore avoid their actual location: mating competition and contest competition; and 2) reasons to minimize using regions within another groups home range: defended space and scramble competition. We therefore aimed to tease apart those two differing components, determining the extent to which the movement patterns of a group were restricted specifically by the location of neighbouring home ranges, rather than through avoidance of another group’s actual location. We then used information on relative group sizes to determine whether those patterns were better explained by defended space or scramble competition.

## Results

### Baseline movement models

The baseline movement model for describing ranging behaviour found that, as predicted by models of optimal foraging^32,33^, the probability of a group visiting a feeding hotspot on a given day decreased with increased distance from each group’s home range centroid (AIC = 2272.75; Fig. 1d; Supplementary Table S2). Controlling for the overall quality of hotspots (capture rate at the hotspot over the entire study period) and the current quality of hotspots (capture rate at the hotspot over 7 days either side of day in question) improved model fit (ΔAIC = 194.56).

### Avoidance models

**Figure 2:**
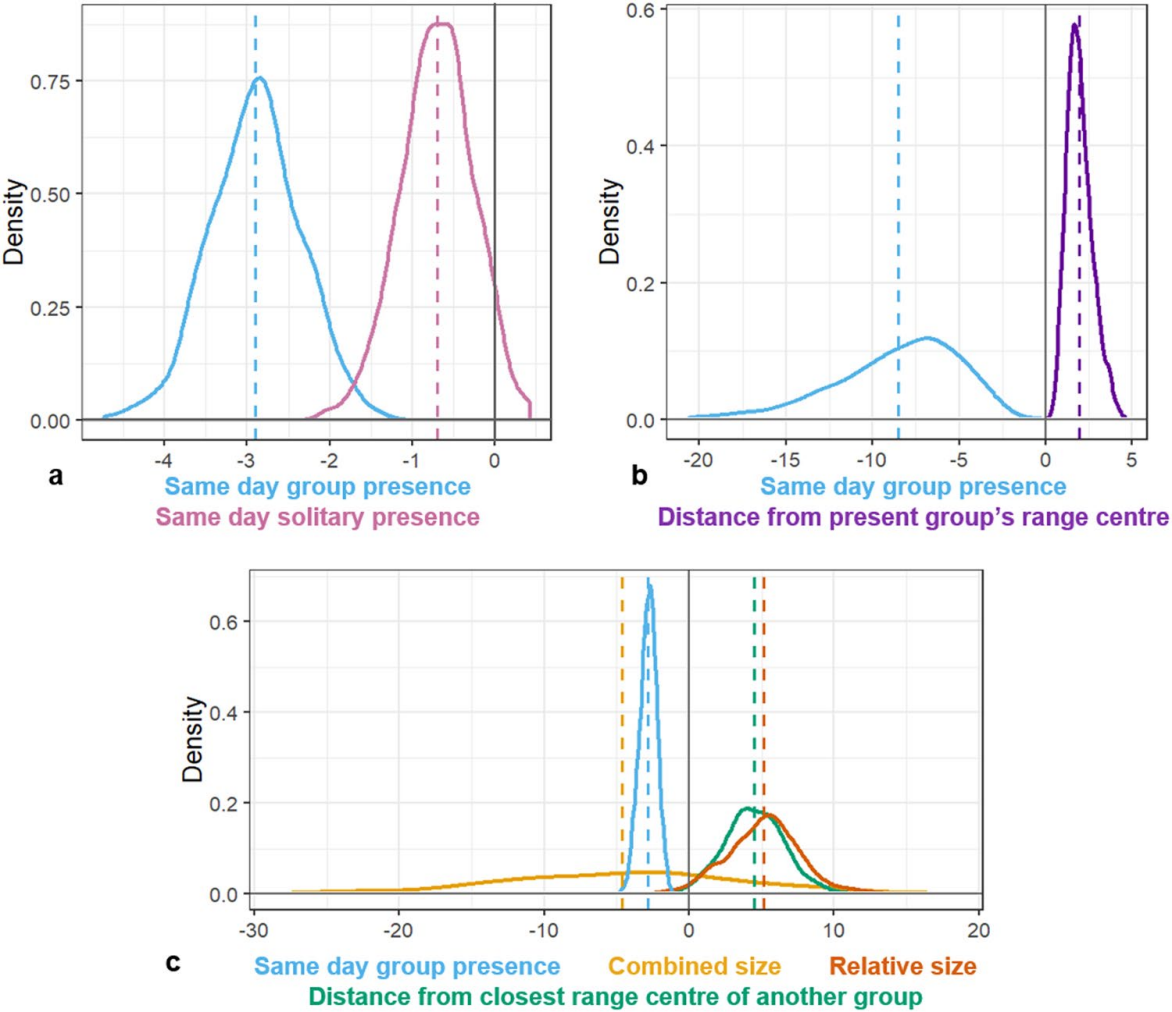
Posterior distributions of model parameters for predicting whether a focal group visited a given site on a given day. (**a**) distributions for the presence of another group (blue) and a solitary male (pink) demonstrate that the presence of another group reduces the likelihood of observing the focal group, but the presence of a solitary does not (overlaps 0). (**b**) Distributions of the presence of other groups (blue) and the distance from those group’s home range centres (purple) demonstrate that the avoidance of other groups decreases with increasing distance from their home range centre. (**c**) Distributions of the presence of groups (blue), the distance from the present group’s home range centre (green), the relative size of groups (group size A ÷ group size B) and the combined group size (group size A+ group size B) demonstrate that groups are more likely to visit when they are comparatively larger than the closest neighbouring group (group B), and when the neighbouring group’s home range is further away.

Building on this baseline model, the model for avoidance of the current location of conspecifics demonstrated that visits to feeding hotspots were less likely when another group visited the same hotspot that day, but not when solitary males visited the hotspot that day (Fig. 2A; Supplementary Table S3). The negative effect of another group’s presence on visit probability decreased with increasing distance from the other group’s home range centroid (Fig. 2B; Supplementary Table S4). In other words, avoidance of another group was greatest near to that group’s home range centroid. This suggests that there may be greater costs to interacting with another group when close to their home range centre, as would be expected if the more central regions of a group’s home range were defended. In addition to the avoidance of groups based on their current location, groups were less likely to visit hotspots the closer they were to another group’s home range centroid. These results demonstrate that avoidance was not based on other groups’ current locations alone but was influenced by their location relative to their neighbours’ home ranges, suggesting that the home ranges of neighbouring groups restrict gorilla movement, in addition to the actual location of conspecifics.

### Reasons for home range avoidance

Two hypotheses were tested as potential explanations for the avoidance of neighbouring groups’ home ranges. Firstly, due to scramble competition, groups may avoid areas central to the home range of another group as they would be less likely to find resources, due to the high activity of that other group in the area^25^. Under this hypothesis, a group’s likelihood of finding adequate resources would be dependent on both the size of the other group using the area and their own size (the groups’ combined size)^34,35^. For example, a group of 3 individuals might still be able to find adequate resources within the home range of a neighbouring group of 20 individuals, and therefore utilize the area, whilst a larger group of 25 might not. Secondly, under a defended space hypothesis, groups could avoid regions used by another group based on the relative threat level they may pose during an aggressive encounter, as a risk avoidance strategy^36–38^. We estimated the relative threat level based on a group’s relative size, as space defence capability should be correlated with mate defence capability and group size^39^. In gorillas, females move groups multiple times and are thought to leave a group if the protective ability of the silverback male declines^40,41^. They are also known to prefer small groups, such that a younger silverback male that has recently developed strong defence capabilities may rapidly accumulate females^42,43^. The home ranges of groups that were relatively larger than the group in question would therefore be expected to be more strongly avoided. In this case, under the example above, the group of 3 individuals would more strongly avoid the home range of a neighbouring group of 20 individuals, whilst the larger group of 25 might not.

The defended space hypothesis fitted our observed data considerably better than the scramble competition hypothesis (defended space model: AIC = 2024.49, scramble competition model: AIC = 2031.23, ΔAIC = 6.74; Table S5), with visits to feeding hotspots more likely when the group in question was relatively larger than the closest neighbouring group, and when the closest neighbouring group’s home range centroid was further away). This model also provided a better fit than a model including both the combined size and relative sizes of groups (Combined model: AIC = 2027.78 ΔAIC = 3.29), in which combined group size overlapped considerably with 0 (Fig. 2C). This demonstrates that the variation explained by combined group size was better explained by relative group size once both predictors were included, suggesting that the defense of space may be a better explanation for the movement patterns observed.

## Discussion

Our results show that gorillas not only avoid their neighbours’ actual location, but factor in the location of their conspecifics’ ranges in the movement decisions they make. The importance of location implies an understanding of the “ownership” of specific geographic regions – usually associated with territoriality^1,10^. The increase in avoidance of other groups closer to their home range centre suggests there may be an increase in the costs of between-group interaction in these regions, consistent with a stronger defensive response from groups when closer to the center of their ranges.

Previous hypotheses for the causes of inter-group avoidance in gorillas have included mating competition and contest competition over food^14,25,29,44,45^. However, these factors alone could not explain the importance of location, as the level of avoidance of other groups would not be expected to vary based on where within each group’s home range the competition was occurring. This pattern would also not be expected if groups were relying on knowledge of their neighbour’s home range location to avoid them when they had limited information on their actual location. In this situation, avoidance should increase with proximity to their neighbours home range centre, but they would not also become better at avoiding those neighbours current location closer to the centre of the home range.

Our model for scramble competition did not appear to explain the observed movement patterns either, as relative size (and therefore the relative defensive capabilities) of the other group and proximity to their home range centre provided a better fit. These movement patterns are therefore consistent with the location-dependent defence of space and the potential for territoriality in western gorillas. However, scramble competition cannot be ruled out entirely as a driver of some of these movement patterns, as the precise foraging strategies used by gorilla groups are not fully known. It is possible that gorilla groups do not take into account their own group’s size in their movement decisions. Nonetheless, these findings emphasize that patterns of space use in gorillas are strongly influenced by the movement patterns of their neighbours, and that they are consistent with the patterns expected if the central regions of gorilla home ranges were defended and therefore avoided by neighbouring groups. Future studies on the location of inter-group aggression are therefore crucial to better understand the potential for territoriality in this species.

Whilst our findings suggest that gorilla groups may have regions of priority or even exclusive use close to their home range centre, groups are known to overlap and even peacefully co-exist in other regions of their ranges^46^. The smaller central home range regions could feasibly be defended by physical aggression, using olfactory cues^47^ or through chest-beating^48^, a commonly observed form of between-group communication in gorillas^26^. We do not suggest that gorillas show the strong forms of territoriality in which neighbours impose a hard boundary on movement, such as in chimpanzees^18^. Rather, our findings support patterns of space sharing and use in gorillas in which there is an understanding of the “ownership” of given areas, the location of neighbouring ranges restrict movement, and the level of inter-group aggression may vary based on the location relative to groups’ home ranges.

The extreme territorial-based violence observed in chimpanzees^18^ has been used as evidence that territorial defense could provide an evolutionary basis for present day warfare^49^, with warfare being a shared evolutionary trait between chimpanzees and humans. However, this warfare likely represents a minority of between-group interactions in human history^50–52^. The more common pattern of interactions may in fact be closer to that suggested in gorillas, with core areas of resident activity dominance and large overlap zones of mutual tolerance^13,50^. The flexible system of defending and sharing space suggested here, combined with growing evidence for long term social bonds between gorilla groups^26,53,54^ and a far more dynamic social system than previously thought^46,55^, implies the presence of a complex social structure in gorillas where between-group interactions are influenced by many factors including social affiliations, kinship and the defence of space. Gorillas may therefore represent a valuable model system for investigating how elements of territoriality can occur simultaneously with affiliative between-group interactions. This will be of particular importance for understanding the social evolution of early human populations, showing both the capacity for extreme territorial-based violence and the between-group affiliations necessary for large-scale cooperation.

## Methods

### Ethics statement

This study was non-invasive and strictly observational, involving no direct contact with gorillas. All data collection followed the relevant guidelines and regulations put in place by the Division of Biological Anthropology at the University of Cambridge and the SPAC Scientific Network and was approved by the Ministère de Recherche Scientifique of Republic of Congo.

### Data collection by camera trapping

Data were collected on wild western lowland gorillas (*Gorilla gorilla gorilla*) non-invasively using automated camera traps deployed across a 60 km^2^ area at the Ngaga Research Site, Republic of Congo (Fig. 1). Camera traps were placed at 36 locations where evidence of non-seasonal root feeding behaviour by gorillas had been observed, primarily surrounding *Maranthes glabra* trees (Supplementary Fig. S1). These locations were chosen as gorillas spent adequate time feeding to enable high rates of group identification compared to a previous gorilla camera trapping project^56^ in which cameras were place on trails (76.9% compared to 22%). Sites represented permanent resources showing minimal seasonal variation in visit frequency (Supplementary Fig. S1). This enabled a five-fold increase in the number of gorilla visits recorded via camera trap used to construct group home ranges, compared to previous methods^56^.

GPS positions of these locations were recorded using a Garmin etrex 30x device. To quantify the rate at which western gorillas visited these root sites, they were monitored over a total of 5403 camera trap days, calculated as the sum of the total number of days that cameras were deployed and functioning at each location, from date of installation to last functional day (last day footage was successfully recorded). Data were collected over 550 days between January 2015 and July 2016. Traps were visited every two weeks to collect the footage and install new batteries. Bushnell Trophy Cam and Reconyx camera traps were used, with one camera at each location, set to record 30 seconds of video footage with each activation.

### Camera trap data processing

Multiple camera trap activations were classed as a single visit when <1 hour had passed between consecutive activations by the same group, or <2 hours had passed between consecutive activations by the same solitary male. This difference in classification was due to the lower rate of camera trap activation from solitary males (as they represented only a single individual and therefore triggered the cameras with lower frequency) compared to higher rates with groups. Group identity was assessed for each camera trap video where possible, resulting in the number of individually identified groups and solitary males specified in supplementary table S1a. Identifications were made from qualitative features of individuals recorded in the camera trap footage^57^. Focal groups (n = 8) included all those that visited 10 or more times in 3 or more locations (Supplementary Table S1b), as a minimum of 3 geographic positions was required to estimate a group’s home range centroid. Individual identity was assessed for each camera trap video including a focal group, where possible. The sizes of focal groups were estimated as the total number of unique individuals identified within the group over the study period (Supplementary Table S1b). Visit data were collated to produce an array indicating for every camera trap day (day a camera trap was functional at a given location) the number of times a given identified group or solitary male had visited that site. Additional columns also included the number of visits from unidentifiable groups and solitary males.

### Statistical methods

Bayesian MCMC algorithms which predicted the likelihood of a focal group visiting a camera trap location on a given day were developed and run in Python version 3.6^58^ as specified in the sample code file. Model selection was done by AIC comparison using ΔAIC and Akaike weight^59^, and by plotting the posterior distributions of model parameters. Posterior distributions from MCMC analysis were plotted in R version 3.5.1^60^ using ggplot2^61^. Posterior values were scaled by the comparative size of their variables (for non-binary variables) to allow clearer comparison and plotting.

### Baseline movement model

Group centroids (home range centres) of the 8 focal groups were estimated from camera trap visit data using an MCMC algorithm that searched for the most likely location of this centroid under the assumption that the frequency with which a site was visited would change as a function of distance from the centroid. Research on gorilla ranging patterns has demonstrated home ranges of between 11 and 18 square kilometres where the majority of a group’s time is spent in a central core area roughly 20–30% of the size of the total home range^24,28–30^. Therefore, models using convex Gaussian and polynomial curves, where visit rate declined slowly close to the centroid but declined faster as distance from the centroid increased were compared with simpler linear distance discounting models (Supplementary Table S2).

$$Linear\,Model\,(A1):\,effect \sim \beta -(\alpha \times distance)$$

$$Gaussian\,Model\,(A2):\,effect \sim \beta +{e}^{-(distanc{e}^{2})/\alpha )}\,$$

$$Polynomial\,Model\,(A3):\,effect \sim \beta +(\alpha \times distance)+-\,(\gamma \times distanc{e}^{2})$$

After identifying the linear model as the best fitting relationship between visit likelihood and distance from a focal group’s centroid, linear models with group-specific β and/or α values were investigated. The best fitting model was one in which β values were the same across all focal groups but α values (gradient of rate of visit decline with distance) were group-specific (Supplementary Table S2; Fig. 1). This model was therefore used as the underlying descriptor for each focal group’s home range, with each focal group assigned an individual centroid (home range centre) and rate of decline in visit probability with distance from that centroid. These individual home range descriptors enabled us to control for any inter-group variation in ranging patterns due group characteristics such as size, composition, habitat type or habituation stage^28,30,31^.

Two further variables were then incorporated in this model to control for qualities of the root sites themselves. ‘Current Quality’ was estimated using the mean number of visits to the root site per day by any other gorilla group or solitary male, over the 7 days either side of the day in question. Only days on which the camera trap was active were included within these means. ‘Current Quality’ was incorporated to control for any seasonal or phenological influences on visit probability at that location e.g. the fruiting of a nearby tree. ‘Overall Quality’ was estimated as the mean number of visits to the root site by any group or solitary male across all days on which a camera trap was functioning at that location. This was incorporated to control for the differing quality of each root site as a resource for gorillas, in addition to the associated travel costs. These controls also accounted for any potential differences in individual camera trap activation rates (either due to the positioning of the cameras or the cameras themselves). Centroids estimated from the baseline movement model were plotted using arcGIS^62^ (Fig. 1).

$$Baseline\,Model:\,effect \sim \beta \,\mbox{--}\,(\alpha \times distance)+\gamma O+\delta C$$

where:

O = *Overall Quality*: popularity of root site with gorillas across the study period

C = *Current Popularity*: popularity of root site with gorillas across 1 week either side of the day in question

### Modeling inter-group dynamics

Visits by other groups and solitary males to sites on a given day were incorporated into models to predict the likelihood of a given focal group visiting that root site on the same day. To determine whether avoidance was occurring based on the current location of other gorillas, the following predictor variables were investigated: (1) visitation to that root site by another group on the same day, (2) visitation to that root site by a solitary male on the same day (3) distance of the root site from the range centre of a group visiting on the same day.

$$Gorilla\,avoidance\,model\,A:effect \sim Baseline\,Model+\zeta G+\varepsilon S$$

$$Gorilla\,avoidance\,model\,B:effect \sim Baseline\,Model+\zeta G+\varepsilon RD$$

where:

S = Visits by solitary gorillas on the same day

G = Visits by other gorilla groups on the same day

RD = distance from the range centre of a group visiting on the same day

Gorilla avoidance model A was investigated using the full dataset including all visits of both known and unknown gorillas. Gorilla avoidance model B was investigated using a smaller sample of visits including only the 8 focal groups (for whom range centres, and therefore, distances from those range centres, could be estimated).

The following predictor variables were then investigated to determine whether avoidance was occurring based on the location of other group’s ranges in addition to those groups’ current locations: (1) distance of the root site from the home range centroid of the nearest neighbouring group (termed the resident group), (2) the relative size of a group compared to the resident group, (3) the combined size of the group and the resident group.

$$Home\,range\,avoidance\,model:effect \sim Baseline\,Model+\zeta G+\varepsilon NC+\eta RS+\theta CS$$

Where:

G = Visits by other gorilla groups on the same day

NC = distance from nearest home range centroid of another group

RS = relative size

CS = combined size

Relative size and combined size of groups were used to investigate whether the location-influenced avoidance of other gorilla groups was better explained by the defence of space within a group’s home range or scramble competition. All combinations of these variables and the distance of the root site from the resident group’s home range centre were tested to identify the model that best fitted the observed data (Supplementary Table S5).

## Supplementary information

Supplementary information.

Supplementary dataset 1.

Supplementary dataset 2.

## Data Availability

All data generated or analysed during this study are included in this published article (and its Supplementary Information files). GPS locations of root sites have been altered such that their position relative to one another remains the same but that their specific location cannot be identified, to prevent issues of poaching and/or theft.
