# Supplementary material for: Western gorilla space use suggests territoriality
Source: Sci Rep. 2020 Mar 12;10:3692. doi: 10.1038/s41598-020-60504-6 (PMC7067784; doi:10.1038/s41598-020-60504-6)
Supplement: Supplementary file 1 — Supplementary information. [file 41598_2020_60504_MOESM1_ESM.pdf]

Supplementary Information for

“Western gorilla space use suggests territoriality”

Robin E Morrison[1,2,3*] Jacob C Dunn[1,4,5], Germán Illera[2], Peter D Walsh[6**], Magdalena Bermejo[2,7**]

[1]Biological Anthropology, University of Cambridge, United Kingdom

[2]SPAC Scientific Field Site Network, GgmbH, Germany

[3]Karisoke Research Center, The Dian Fossey Gorilla Fund, Rwanda

[3]Behavioural Ecology Research Group, Anglia Ruskin University, United Kingdom

[4]Department of Cognitive Biology, University of Vienna, Austria

[5]Apes Incorporated, USA

[6]Ecology and Environmental Sciences, University of Barcelona, Spain

*Correspondence to robinemilymorrison@gmail.com

[**]Joint Senior Author

Files:

Visit Data Set (SI File 1): For all identified groups, and for unidentified groups and solitaries, across all camera trap days at all locations, the number of times they visited that day.

Hotspot Locations (SI File 2): Relative locations of hotspots at which camera traps were deployed (decimal longitude and latitude values relative to the lowest longitude and latitude values at which a camera was deployed).

Supplementary Figures:

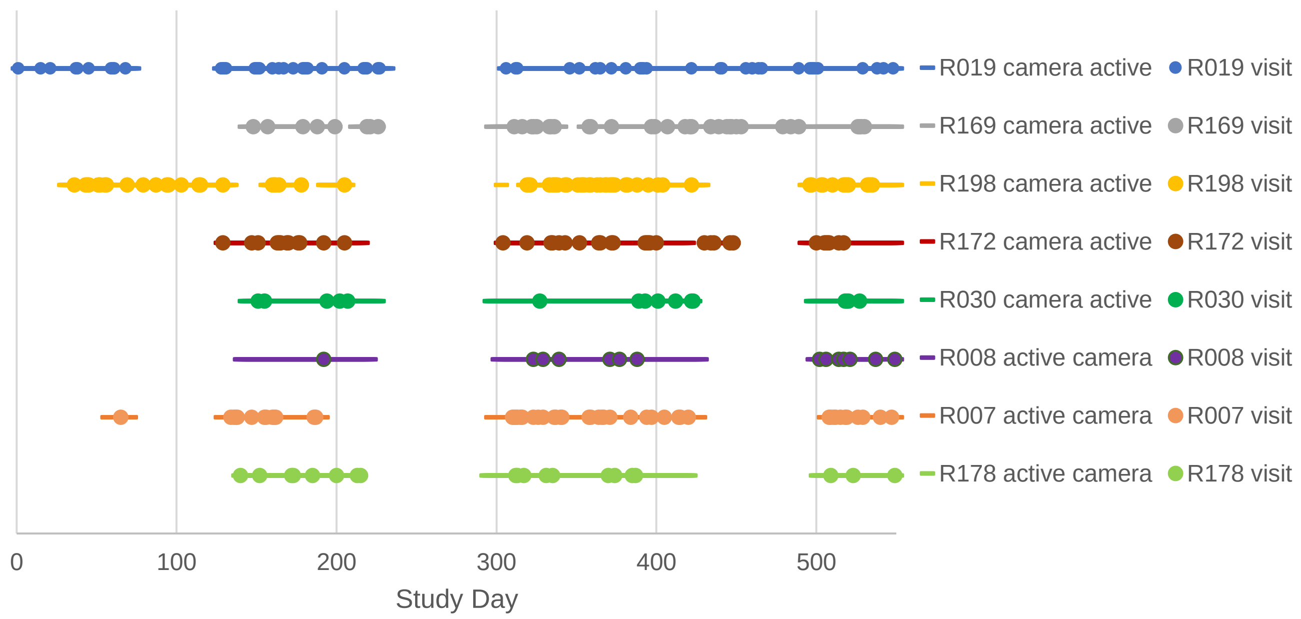

Figure S1. The distribution of visits by gorilla groups and solitary males to the 8 best monitored locations across the study period demonstrates that there was no clear overall seasonal variation in the use of “hotspot” root site locations. Furthermore, visits to individual hotspots were fairly evenly spread across their sampling periods without the clear seasonal variation expected for food sources such as fruiting trees that come in and out of season.

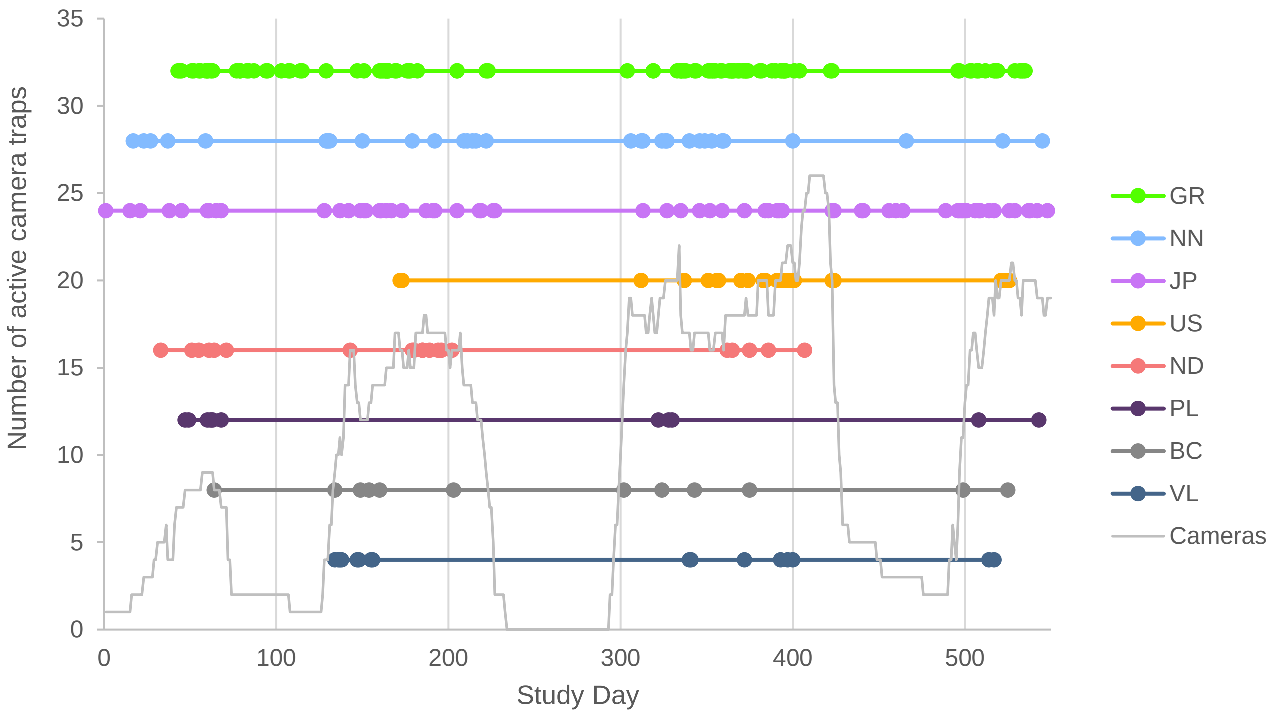

Figure S2. Distribution of recorded visits by each focal group across the study period demonstrates that focal groups were observed in a roughly even manner across the study period. Detection frequencies did not appear to follow any overall seasonal variation although, as expected were strongly influenced by the number of active camera traps.

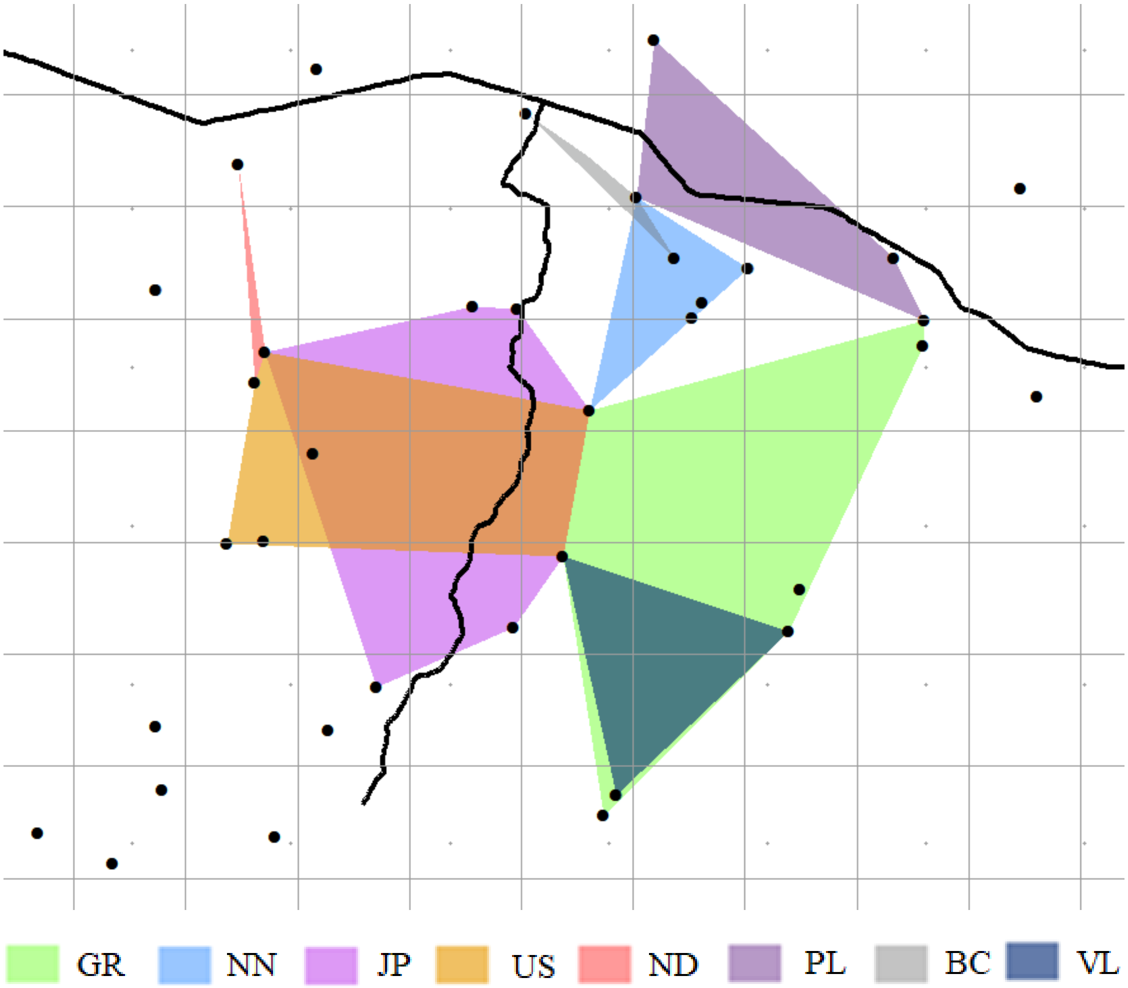

Figure S3. Minimum convex polygon ranges for the eight focal groups, from presence/absence at camera trap locations (black dots), with 1km^2 grid overlaid. Roads indicated by black lines. This method of home range estimation predicts only the outer limits of the home range and requires a considerable sample size in order to produce a realistic home range. It also produces poor estimates when a group is not sampled across their full range. This map provides useful information on where gorilla groups were sampled but the home range centroids in Figure 1B provide more accurate estimates of the focal gorilla groups' home range locations by incorporating information on the relative frequency of capture at each location.

Supplementary Tables:

Table S1. A) Total number of identified groups and solitaries, and the number of visits by each category type. B) Number of visits and the number of locations those visits took place at for each focal group.

A

	**Number of**	**Visits by**
Known groups	24	386
Known Solitaries	6	51
Unknown groups	-	90
Unknown solitaries	-	41
**Total**		**568**

B

**Group**		**Visits**	**Locations**	**Size**
1	GR	104	7	22
2	JP*	65	7	19
3	NN*	32	6	15
4	US	22	6	8
5	ND	20	3	12
6	VL	16	3	18
7	BC	12	3	3
8	PL*	11	5	16

*groups undergoing habituation

Table S2. Model comparison of baseline distance discounting by focal groups. Best model fit (lowest AIC) indicated in bold.

Model	Group-specific α	Group-specific β	AIC
**A1 Linear**	No	No	2349.90
**A2 Gaussian**	No	No	3431.356
**A3 Polynomial**	No	No	2475.792
**A1 Linear**	No	Yes	2494.93*
**A1 Linear**	Yes	No	**2272.75**
**A1 Linear**	Yes	Yes	2422.074*

*could not converge

Table S3. Posterior variable values (mean with 95% confidence intervals in brackets) for all combinations of variables in the Gorilla Avoidance Model A and their AIC scores. Best fitting model indicated in bold.

Model	Solitary presence (ε)	Group presence (ζ)	AIC	Akaike weight
Baseline Model + ζG	-0.51 (-1.47, 0.32)	-	2080.98	4.05E-09
Baseline Model + εS	-	-2.87 (-4.02,-1.81)	**2042.33**	**0.612**
Baseline Model + ζG + εS	-0.70 (-1.60, 0.12)	-2.90 (-3.98,-1.83)	2043.24	0.388

Table S4. Posterior variable values (mean with 95% confidence intervals in brackets) for Gorilla Avoidance Model B and AIC scores when predicting group presence based on the same day presence of only other focal groups (as distance from their centroid is known). Best fitting model indicated in bold.

Model	Group presence on day (ζ)	Avoidance with distance from other group's range centroid (ε)	AIC
Baseline Model + ζG	-0.83 (-1.98, 0.05)	**-**	2260.25
Baseline Model + ζG + εRD	-8.50 (-16.97, -3.08)	1.95 (0.78, 3.65)	**2244.38**

Table S5. Posterior variable values (mean with 95% confidence intervals in brackets) for all combinations of additional variables in the home range avoidance model with their AIC scores and Akaike weights. Best fitting model indicated in bold.

**Group presence on day (ζ)**	**Distance from centroid of another group (η)**	**Relative size (territoriality)**	**Combined size (scramble competition)**	**AIC**	**Akaike weight**
-2.82 (-4.00, -1.78)	4.80 (1.39, 8.54)	-	-	2036.68	0.002
-2.80 (-3.95, -1.73)	3.22 (-0.49, 7.02)	6.66 (3.85, 965)	**-**	**2024.49**	**0.696**
-2.79 (-3.91, -1.80)	4.72 (0.61, 8.59)	-	-18.11 (-29.81, -5.61)	2031.25	0.024
-2.83 (-4.01, -1.70)	4.53 (0.48, 8.78)	5.15 (0.15, 10.50)	-4.63 (-23.85, 12.06)	2027.78	0.134
-2.89 (-4.21, -1.85)	-	6.16 (3.36, 9.00)	-	2028.35	0.101
-2.92 (-4.10, -0.10)	-	-	-15.82 (-27.80, -2.78)	2037.07	0.001
-2.90 (-4.00, -1.89)	-	6.16 (2.57, 9.83)	-1.28 (-19.15, 13.71)	2030.12	0.042

Example python code for MCMC analysis

```
import numpy as np
import random
import os
replicates = 200000
burnin = 500
autocorrelation = 200

#set priors
alpha = [25.65, 25.65, 25.65, 25.65, 25.65, 25.65, 25.65, 25.65]
beta = -0.58
gamma = 0.01
delta = 0.01

#mean X and Y coordinates of hotspots set as centroid priors
CentroidX = [0.039, 0.039, 0.039, 0.039, 0.039, 0.039, 0.039, 0.039]
CentroidY = [0.032, 0.032, 0.032, 0.032, 0.032, 0.032, 0.032, 0.032]

like = 0
for i in range(numdays):
   for j in range(numgroups):
            # distance of centroid from hotspot
            distance = ((CentroidX[j] - HotspotX[i])**2 + (CentroidY[j] - HotspotY[i])**2) ** 0.5
            # Model
            effect = beta - (alpha[j] * distance) + (gamma * OverallQuality[i,j]) + (delta * CurrentQuality[i,j])
            if VisitData[i,j]==1:
             like = like + np.log(np.exp(effect) / (1 + np.exp(effect)))
            else:
             like = like + np.log(1 - (np.exp(effect) / (1 + np.exp(effect))))
SupportDist = open("SupDist.txt", "w")

for z in range(replicates):
   newalpha[0] = alpha[0] + 0.5 * (2 * random.random() - 1)
   newalpha[1] = alpha[1] + 0.5 * (2 * random.random() - 1)
   newalpha[2] = alpha[2] + 0.5 * (2 * random.random() - 1)
   newalpha[3] = alpha[3] + 0.5 * (2 * random.random() - 1)
   newalpha[4] = alpha[4] + 0.5 * (2 * random.random() - 1)
   newalpha[5] = alpha[5] + 0.5 * (2 * random.random() - 1)
   newalpha[6] = alpha[6] + 0.5 * (2 * random.random() - 1)
   newalpha[7] = alpha[7] + 0.5 * (2 * random.random() - 1)
   newbeta = beta + 0.1 * (2 * random.random() - 1)
   newgamma = gamma + 0.2 * (2 * random.random() - 1)
   newdelta = delta + 0.2 * (2 * random.random() - 1)
   NewCentroidX[0] = CentroidX[0] + 0.00005 * (2 * random.random() - 1)
   NewCentroidX[1] = CentroidX[1] + 0.00005 * (2 * random.random() - 1)
   NewCentroidX[2] = CentroidX[2] + 0.00005 * (2 * random.random() - 1)
   NewCentroidX[3] = CentroidX[3] + 0.00005 * (2 * random.random() - 1)
```

```
NewCentroidX[4] = CentroidX[4] + 0.00005 * (2 * random.random() - 1)
NewCentroidX[5] = CentroidX[5] + 0.00005 * (2 * random.random() - 1)
NewCentroidX[6] = CentroidX[6] + 0.00005 * (2 * random.random() - 1)
NewCentroidX[7] = CentroidX[7] + 0.00005 * (2 * random.random() - 1)
NewCentroidY[0] = CentroidY[0] + 0.00005 * (2 * random.random() - 1)
NewCentroidY[1] = CentroidY[1] + 0.00005 * (2 * random.random() - 1)
NewCentroidY[2] = CentroidY[2] + 0.00005 * (2 * random.random() - 1)
NewCentroidY[3] = CentroidY[3] + 0.00005 * (2 * random.random() - 1)
NewCentroidY[4] = CentroidY[4] + 0.00005 * (2 * random.random() - 1)
NewCentroidY[5] = CentroidY[5] + 0.00005 * (2 * random.random() - 1)
NewCentroidY[6] = CentroidY[6] + 0.00005 * (2 * random.random() - 1)
NewCentroidY[7] = CentroidY[7] + 0.00005 * (2 * random.random() - 1)

newlike = 0
for i in range(numdays):
    for j in range(numgroups):
        distance = ((NewCentroidX[j] - HotspotX[i]) ** 2 + (NewCentroidY[j] - HotspotY[i]) ** 2) ** 0.5
        effect = newbeta - (newalpha[j] * distance)+ (newgamma * OverallQuality[i,j]) + (newdelta *
        CurrentQuality[i,j])
        if VisitData[i,j]==1:
            newlike = newlike + np.log(np.exp(effect) / (1 + np.exp(effect)))
        else:
            newlike = newlike + np.log(1 - (np.exp(effect) / (1 + np.exp(effect))))

if random.random()<(np.exp(newlike-like)):
    like=newlike
    alpha[0]=newalpha[0]
    alpha[1] = newalpha[1]
    alpha[2] = newalpha[2]
    alpha[3] = newalpha[3]
    alpha[4] = newalpha[4]
    alpha[5] = newalpha[5]
    alpha[6] = newalpha[6]
    alpha[7] = newalpha[7]
    beta=newbeta
    gamma=newgamma
    delta=newdelta
    CentroidY[0]=NewCentroidY[0]
    CentroidY[1] = NewCentroidY[1]
    CentroidY[2] = NewCentroidY[2]
    CentroidY[3] = NewCentroidY[3]
    CentroidY[4] = NewCentroidY[4]
    CentroidY[5] = NewCentroidY[5]
    CentroidY[6] = NewCentroidY[6]
    CentroidY[7] = NewCentroidY[7]
    CentroidX[0] = NewCentroidX[0]
    CentroidX[1] = NewCentroidX[1]
    CentroidX[2] = NewCentroidX[2]
```

```
        CentroidX[3] = NewCentroidX[3]
        CentroidX[4] = NewCentroidX[4]
        CentroidX[5] = NewCentroidX[5]
        CentroidX[6] = NewCentroidX[6]
        CentroidX[7] = NewCentroidX[7]

    if z >= burnin:
        if z%autocorrelation==0:

SupportDist.write('{}\t{}\t{}\t{}\t{}\t{}\t{}\t{}\t{}\t{}\t{}\t{}\t{}\t{}\t{}\t{}\t{}\t{}\t{}\t{}\t{}\t{}\t{}\t{}\t{}\
t{}\t{}\t{}\t{}\t{}\n'.format(z, like,
alpha[0],alpha[1],alpha[2],alpha[3],alpha[4],alpha[5],alpha[6],alpha[7], beta, gamma, epsilon, zeta, eta,
theta, CentroidX[0], CentroidY[0],CentroidX[1], CentroidY[1], CentroidX[2], CentroidY[2],CentroidX[3],
CentroidY[3],CentroidX[4], CentroidY[4],CentroidX[5], CentroidY[5],CentroidX[6],
CentroidY[6],CentroidX[7], CentroidY[7]))
SupportDist.close()
```
